# Seasonal associations of climatic drivers and malaria in the highlands of Ethiopia

**DOI:** 10.1186/s13071-015-0954-7

**Published:** 2015-06-24

**Authors:** Alemayehu Midekisa, Belay Beyene, Abere Mihretie, Estifanos Bayabil, Michael C. Wimberly

**Affiliations:** Geospatial Sciences Center of Excellence (GSCE), South Dakota State University, Brookings, SD USA; Amhara Regional Health Bureau, Bahir Dar, Ethiopia; Health Development and Anti-Malaria Association, Addis Ababa, Ethiopia

**Keywords:** Malaria, Epidemic, Climate, Weather, Early warning, Mosquito

## Abstract

**Background:**

The impacts of interannual climate fluctuations on vector-borne diseases, especially malaria, have received considerable attention in the scientific literature. These effects can be significant in semi-arid and high-elevation areas such as the highlands of East Africa because cooler temperature and seasonally dry conditions limit malaria transmission. Many previous studies have examined short-term lagged effects of climate on malaria (weeks to months), but fewer have explored the possibility of longer-term seasonal effects.

**Methods:**

This study assessed the interannual variability of malaria occurrence from 2001 to 2009 in the Amhara region of Ethiopia. We tested for associations of climate variables summarized during the dry (January–April), early transition (May–June), and wet (July–September) seasons with malaria incidence in the early peak (May–July) and late peak (September–December) epidemic seasons using generalized linear models. Climate variables included land surface temperature (LST), rainfall, actual evapotranspiration (ET), and the enhanced vegetation index (EVI).

**Results:**

We found that both early and late peak malaria incidence had the strongest associations with meteorological conditions in the preceding dry and early transition seasons. Temperature had the strongest influence in the wetter western districts, whereas moisture variables had the strongest influence in the drier eastern districts. We also found a significant correlation between malaria incidence in the early and the subsquent late peak malaria seasons, and the addition of early peak malaria incidence as a predictor substantially improved models of late peak season malaria in both of the study sub-regions.

**Conclusions:**

These findings suggest that climatic effects on malaria prior to the main rainy season can carry over through the rainy season and affect the probability of malaria epidemics during the late malaria peak. The results also emphasize the value of combining environmental monitoring with epidemiological surveillance to develop forecasts of malaria outbreaks, as well as the need for spatially stratified approaches that reflect the differential effects of climatic variations in the different sub-regions.

## Background

The impact of interannual climate fluctuations on vector-borne diseases, especially malaria, has received considerable attention in the scientific literature [1–4]. The influences of climatic variations on malaria risk can be especially pronounced in semi-arid and high-elevation areas such as the highlands of East Africa [3, 5]. Cool temperatures and seasonally dry conditions limit malaria transmission in the highlands, and as a result population-level immunity is typically low [6]. However, epidemics can still occur in response to abnormally warm temperatures and wet conditions. Multiple large-scale epidemics of malaria have been documented in the East African highlands in recent decades [7–9]. Malaria epidemics in these regions can create a large disease burden with high rates of mortality and morbidity across all age groups. Because of the devastating nature of these epidemics, understanding the effects of short-term climate variations on malaria transmission is an important step toward allowing public health decision makers to plan intervention strategies more effectively.

Although temporal variations in malaria transmission are influenced by multiple factors including land use change, public health interventions, and other socioeconomic determinants, the role of climate is crucial [10]. For example, an earlier study reported associations of interannual variation in temperature and rainfall with malaria outpatient cases in the East African highlands including Ethiopia, Kenya, and Uganda [3]. Strong associations of temperature and rainfall with malaria cases were also found in the highlands of Ethiopia [11]. Temperature influences the development and survival rates of both the anopheline mosquito vectors and the *Plasmodium* parasites that cause malaria; as a result, malaria transmission rates tend to increase with temperature up to a threshold level [12, 13]. Higher temperature will increase the feeding frequency of female adult mosquitoes, which can increase the probability of transmitting the malaria parasite to uninfected human hosts [14]. The duration of the sporogonic cycle, in which the parasite develops inside the mosquito, also shortens as temperature increases up to an optimum level [13, 15]. In addition, the longevity of mosquitoes is sensitive to temperature, and there are threshold temperatures above which mosquito mortality increases and minimum temperatures below which mosquitoes become inactive [14]. Anopheline mosquitoes also need surface water to complete their life cycles. Rainfall influences malaria transmission by supplying water to create aquatic habitats, although excessive rainfall may flush breeding sites and cause mortality of mosquito larvae [16–19].

Most previous studies that examined the link between climate and malaria have reported lagged associations of climate variables such as temperature and rainfall with malaria cases over time periods ranging from weeks to months [3, 6, 11, 20]. These associations are based on the assumption that short term lags reflect the time required following a climatic anomaly for mosquitoes to develop to adulthood, acquire and transmit a malarial infection, and for symptoms to arise in the human host [11]. However, mosquito populations, mosquito infection rates, and the resulting risk of malaria in humans may also respond to longer-term cumulative effects of climatic fluctuations on mosquito populations and malaria infection levels across multiple seasons. Although malaria transmission during the wet season can be limited by the cooler temperatures associated with summer rains, there is also a potential for carryovers of parasites in the human population to the subsequent epidemic season. Long-term asymptomatic carriage of *Plasmodium* parasites, especially in regions of seasonal transmission, is common [21–23]. Therefore, there is a possibility that lagged effects of climate variables will extend across multiple seasons. For example, a study in the highlands of western Kenya found evidence of a “ripple effect” in which November–January rainfall was correlated with the number of February–April malaria cases, which was in turn correlated with the number of cases during the main malaria season in May–December [1].

Here, we test this conceptual model in the highlands of the Amhara region of Ethiopia by assessing the effects of satellite-derived climate variables summarized during the dry (January–April), early transition (May–June), and wet (July–September) seasons with malaria incidence in the early (May–July) and late (September–December) peak transmission seasons. Districts in western Amhara experience longer and heavier rainy seasons and higher intra-annual variability in temperature, whereas districts in eastern Amhara experience shorter rainy seasons and less intra-annual variability in temperature. Because different factors may influence malaria incidence under each of these climate regimes [9], we analyzed each sub-region separately. We hypothesized that malaria occurrence during the early peak season would be associated with the cumulative effects of climatic conditions during the preceding dry and early transition seasons. We further hypothesized that malaria cases during the early peak season would be correlated with malaria cases during the subsequent late peak season, reflecting the carryover of parasites in the human population. Because of this linkage, and because cooler temperatures during the wet season will likely reduce malaria transmission, malaria during the late peak season should have stronger associations with dry and early transition season climatic variables than wet season climatic variables. We also hypothesized that including early peak malaria incidence as an independent variable in models of late peak season malaria would improve their fit compared to models based on climatic variables alone. Finally, we hypothesized that temperature would be a more important predictor of malaria incidence in the cooler and wetter western districts, whereas moisture would be more important in the warmer and drier eastern districts. We addressed these hypotheses by using remote sensing products derived from multiple earth observation satellites and analyzing the seasonal associations of malaria incidence with climate conditions.

## Methods

### Study area

**Fig. 1:**
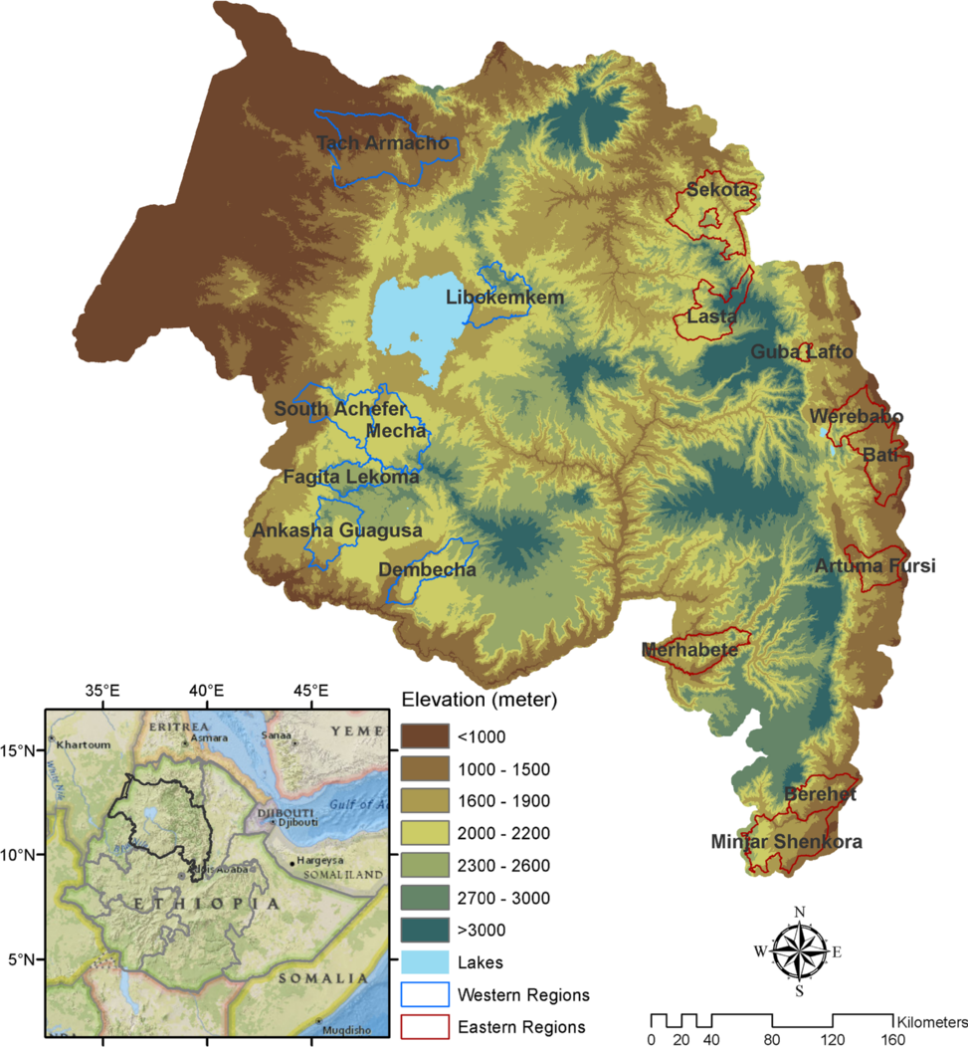
Map of the study area in the Amhara region of Ethiopia showing the seven western districts and nine eastern districts used in the analysis

The study area, the Amhara region, is located in the northwestern and north central parts of Ethiopia and lies within 9 ° and 13 °45 N and 36 ° and 40 °30 E (Fig. 1). The Amhara region has 11 administrative zones and has a total area of approximately 170,000 km^2^. Elevation ranges from 506 to 4517 m above sea level. Mean annual rainfall ranges from 770 to 2000 mm while average annual air temperature ranges from 16 °C in the summer to 27 °C in the dry season. In this highland region, malaria is characterized by unstable transmission, and outbreaks of malaria can cause high morbidity and mortality because the human population lacks immunity to the pathogen [24, 25]. For example, the most recent regional malaria epidemics occurred from 2003 to 2005 [25, 26]. Since that time, malaria outbreaks have generally been smaller and more localized [9]. *Anopheles arabiensis* is the principal malaria vector in the Amhara region [27], and *Plasmodium falciparum and Plasmodium vivax* are the major malaria parasites in the region [28]. Ethiopia has a national malaria control program, including the Amhara region, which consists of the distribution of free long-lasting insecticidal nets (LLINs), targeted indoor residual spraying (IRS), rapid diagnostic tests (RDTs), and treatment with artemisinin combination therapy [28]. Malaria transmission is seasonal in the Amhara region following seasonal patterns of climatic factors; the two main malaria transmission seasons occur in May–July following the end of the dry season and in September–December following the end of the wet season. Previous studies have documented regional spatial synchrony of malaria outbreaks [9], and others have found lagged associations of climate variables and malaria in the region with time lags ranging from 1 to 3 months [20, 29].

### Environmental data

We used rainfall estimates from the Tropical Rainfall Measuring Misssion (TRMM) with a spatial resolution of 0.25° × 0.25°. The TRMM preciptation (3B42) product was used to compute seasonal total rainfall (mm) for each district. We used the 8-day land surface temperature (LST) product (MOD11A2) from the Moderate Resolution Imaging Spectroradiometer (MODIS) insrtument, on the Terra satellite, at a 1 km spatial resolution to calculate seasonal mean LST for each district. We also computed the 8-day enhanced vegetation index (EVI) using surface reflectance in the blue, red and near-infrared bands from the MODIS Nadir BRDF-Adjusted Reflectance (NBAR) product (MCD43B4) at a 1 km spatial resolution. All these remote sensing data were processed using the EASTWeb software developed at the Geospatial Sciences Center of Excellence, South Dakota State University [30]. In addition, we obtained satellite-derived 8-day actual evapotranspiration (ET) based on Simplified Surface Energy Balance Operational (SSEBop) model [31]. The SSEBop is an operational ET product that uses MODIS and Global Data Assimilation System (GDAS) data and is available through the U.S. Geological Survey. We summarized these satellite-derived climate variables for each district using the zonal statistics method over the 2001–2009 periods. We then calculated seasonal summaries for the dry (day of year (DOY) 1–120), early transition (DOY 121–180), and wet (DOY 181–273) seasons. We computed seasonal means for LST and EVI and seasonal totals for ET and rainfall for each of the three seasons. We also included the district-level percent of herbaceous wetlands, which serve as a natural reservoir for the malaria vector, in each district as an independent variable in all the models. Wetland cover was computed from a land cover map for the Amhara region derived from spectral and topographic indices from Landsat TM/ETM+ and the Shuttle Radar Topography Mission (SRTM) respectively [32].

### Malaria surveillance data

**Fig. 2:**
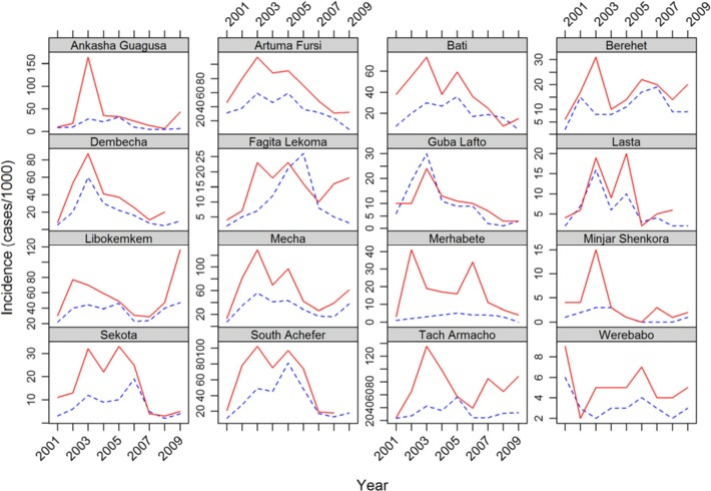
Interannual variability in early peak (*blue dashed lines*) and late peak (*red solid lines*) malaria incidence from 2001 to 2009 for 16 districts in the Amhara region. Locations of the 16 districts are referenced in Fig. 1

**Fig. 3:**
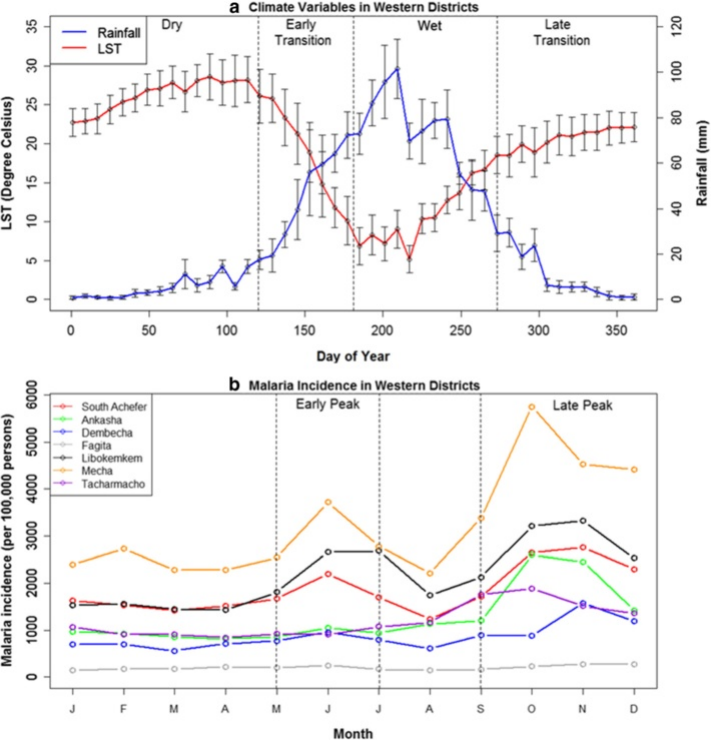
**a** Seasonal patterns of satellite-derived estimates of rainfall and land surface temperature (LST) averaged over the 2001–2009 period for the seven western districts in the Amhara region, Ethiopia. **b** Seasonal pattern of malaria incidence (per 100,000) in the seven western districts. The *vertical bars* represent one standard deviation and show variability among districts. The seven western districts are referenced in Fig. 1

**Fig. 4:**
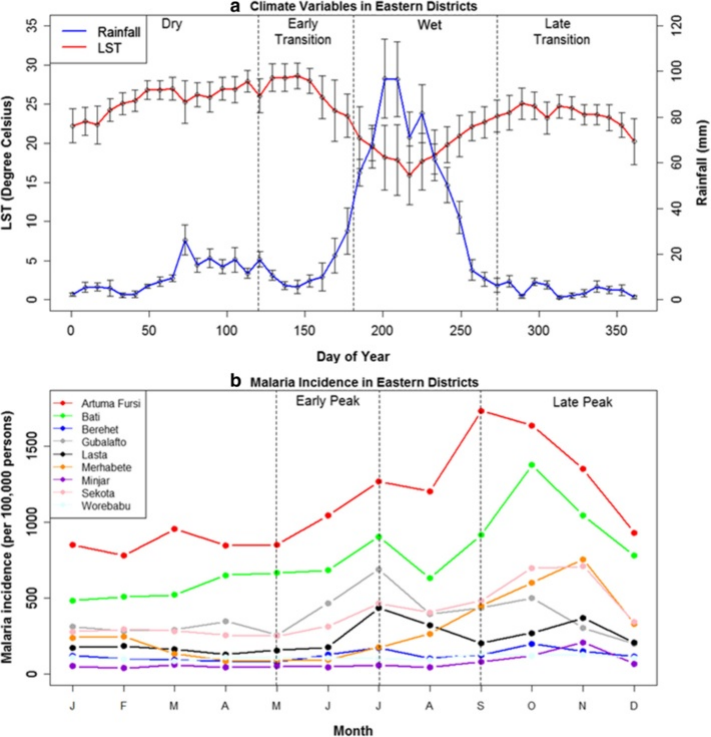
**a** Seasonal pattern of satellite-derived estimates of rainfall and land surface temperature (LST) averaged over the 2001–2009 periods for the nine eastern districts. **b** Seasonal pattern of malaria incidence (per 100,000) in the nine eastern districts. The *vertical bars* represent one standard deviation and show variability among districts. The nine eastern districts are referenced in Fig. 1

We used clinically-diagnosed outpatient malaria cases from 16 districts in the Amhara region. Counts of malaria cases including all age groups and all *Plasmodium* species for 2001–2009 were collected from multiple district (*woreda*) health offices and the Federal Ministry of Health, Public Health Emergency Management office by the Health, Development and Anti-Malaria Association, an Ethiopian NGO [9]. Data were reported using the integrated disease surveillance and response (IDSR) summary forms that are routinely used for surveillance of malaria and other infectious diseases [33]. Malaria incidence was calculated by dividing the total number of clinically-diagnosed outpatient malaria cases by the total population of each district based on the 2007 national population census of Ethiopia [34] and multiplying by 1000 to provide malaria incidence per 1000 persons. Malaria incidence over 2001–2009 was calculated for the two main malaria transmission seasons: early peak (May–July) and late peak (September–December). Interannual variability in early and late peak malaria incidence for each district in Fig. 2. We selected the 16 districts for analysis based on availability of the malaria surveillance data for the entire 2001–2009 study period. We divided these districts into western districts (seven districts) and eastern districts (nine districts) based on observed climatological patterns (Figs. 1, 3a and 4a).

### Statistical methods

We conducted exploratory analysis to visualize the seasonal patterns of rainfall and LST in relation to malaria incidence in order to highlight differences in climatology and malaria epidemiology between the two regions. We used a negative binomial generalized linear model (GLM) to test the seasonal associations of malaria incidence and climatic drivers. Separate models were developed for the western and eastern districts. Early peak and late peak season malaria incidence were used as response variables, the natural logarithm of total population was used to account for spatial variability in population, and seasonal climatic variables were used as independent variables. In all the models, we also included percent wetlands as an independent variable to control for the effect of spatial variability in local hydrology [32]. We used a square-root transformation of the percent wetlands to minimize the influences of outlying values and meet the statistical assumptions of the GLM model.

We used a multimodel inference approach to determine the best models for dry, early transition and wet season climate effects on early and late peak malaria. For parsimony, we considered seven candidate models that included all possible combinations of the temperature variable (LST), and one of the three moisture indicators (rainfall, ET, and EVI) (Tables 1 and 2). The models were assessed using Akaike Information Criterion (AIC) scores, which measure the relative goodness of model fit. Additionally, we used Akaike weights to evaluate these models [35]. Akaike weights measure the probability that a model is the best model given the data and the set of candidate models [35]. Lower AIC statistics and higher Akaike weights indicated better model fit.

**Table 1:** Early peak season malaria model results in the western and eastern districts

		Western districts			Eastern districts		
Season	Model rank	Variables	AIC	Akaike weight	Variables	AIC	Akaike weight
Dry	1	LST, ET	508.6	0.44	ET	552.8	0.58
	2	LST, EVI	509.7	0.26	LST, ET	553.6	0.39
	3	LST	510.2	0.20	LST, EVI	559.0	0.03
	4	LST, Rainfall	512.1	0.08	EVI	560.9	0.01
	5	EVI	514.6	0.02	LST, Rainfall	575.3	0.00
	6	ET	517.7	0.00	Rainfall	577.3	0.00
	7	Rainfall	521.0	0.00	LST	577.9	0.00
Early	1	LST	509.5	0.43	LST, ET	553.0	0.85
	2	LST, Rainfall	510.9	0.22	ET	557.5	0.09
	3	LST, ET	511.4	0.17	LST, EVI	558.4	0.06
	4	LST, EVI	511.5	0.16	EVI	565.3	0.00
	5	EVI	516.3	0.01	LST	573.5	0.00
	6	ET	518.4	0.01	LST, Rainfall	574.5	0.00
	7	Rainfall	521.1	0.00	Rainfall	578.1	0.00

*LST* land surface temperature, *EVI* enhanced vegetation index, *ET* actual evapotranspiration

**Table 2:** Late peak season malaria model results in the western and eastern districts

		Western districts			Eastern districts		
Season	Model rank	Variables	AIC	Akaike weight	Variables	AIC	Akaike weight
Dry	1	LST	568.1	0.34	LST, EVI	634.0	0.64
	2	LST, ET	568.5	0.28	LST, ET	635.2	0.35
	3	LST, EVI	569.2	0.19	ET	644.2	0.00
	4	LST, Rainfall	569.6	0.16	EVI	648.2	0.00
	5	EVI	574.2	0.02	LST, Rainfall	649.5	0.00
	6	Rainfall	575.0	0.01	LST	656.3	0.00
	7	ET	576.5	0.01	Rainfall	662.0	0.00
Early	1	LST	554.7	0.38	LST, EVI	635.9	0.66
	2	LST, ET	555.0	0.33	LST, ET	637.2	0.34
	3	LST, Rainfall	556.5	0.15	LST	649.2	0.00
	4	LST, EVI	556.7	0.14	LST, Rainfall	650.8	0.00
	5	ET	564.1	0.00	ET	653.7	0.00
	6	EVI	569.0	0.00	EVI	654.2	0.00
	7	Rainfall	576.3	0.00	Rainfall	663.4	0.00
Wet	1	Rainfall	572.1	0.56	LST, ET	639.0	0.79
	2	LST, Rainfall	573.6	0.26	LST, EVI	642.4	0.14
	3	ET	576.2	0.07	LST, Rainfall	645.1	0.04
	4	EVI	577.1	0.05	LST	645.9	0.03
	5	LST, ET	578.1	0.03	ET	656.5	0.00
	6	LST, EVI	579.1	0.02	EVI	660.0	0.00
	7	LST	579.3	0.02	Rainfall	664.4	0.00

*LST* land surface temperature, *EVI* enhanced vegetation index, *ET* actual evapotranspiration

We used Spearman’s rank correlation to test the association between early and late peak season malaria incidence. The correlation analysis was based on malaria incidence in each of the two sub regions for 2001–2009. We calculated malaria incidence rates by summing the annual numbers of early and late peak malaria cases over the districts in each sub region and dividing by the total population summed over the districts in each sub region. A separate Spearman’s rank correlation was also computed for each of the 16 sub-districts. We tested whether including early peak malaria incidence as an independent variable improved the fit of the best climatic models of late peak malaria by comparing model 1 (with early peak malaria incidence as an independent variable) with model 2 (without early peak malaria incidence as an independent variable) using AIC as described previously. We conducted all statistical modeling in the R environment for statistical computing [36].

## Results

The two sub-regions of the Amhara region showed different climatic conditions. The eastern districts had shorter summer rains and less intra-annual variation in temperature, whereas the western districts had longer summer rains and higher intra-annual variation in temperature (Figs. 3a and 4a). Moreover, the eastern districts experienced an earlier, smaller peak of rainfall from March–May. Overall, the western districts were characterized by wetter and colder climates while the eastern districts had drier and warmer climates. The malaria incidence data showed two distinct peaks during the early and late peak malaria transmission periods (Figs. 3b and 4b); the former matched with the start of the wet season (summer rains) while the latter followed the end of the wet season. In the eastern districts the early peak malaria season began sooner than in the western districts. The western districts had a higher malaria incidence overall.

Table 1 summarizes the AIC scores and weights for the seven models for early peak malaria in the two sub-regions based on climatic variables from the dry and early transition seasons. In the dry season models, LST had a strong influence and adding moisture variables did not substantially improve model fit in the western districts, whereas moisture variables had the strongest influence and adding LST did not improve model fit in the eastern districts. In the early transition season models, LST had the strongest influence and adding moisture variables did not improve model fit in the western districts, whereas both LST and moisture indicators contributed to the fit of the best model in the eastern districts. Overall, for the models of early peak season malaria, LST had the strongest influence in the western districts, whereas moisture variables were more important in the eastern districts (Table 1). There was no substantive difference (delta AIC values <2) between the best models based on dry season climate and early transition season climate in either region.

Table 2 summarizes the seven models for late peak season malaria in the two sub-regions based on climatic variables from the dry, early transition, and wet seasons. In the dry and early transition season models, LST had the strongest influence in the western districts and adding moisture variables did not substantially improve model fit, whereas both LST and moisture contributed to model fit in the eastern districts. In the wet season models, moisture variables had the strongest influence and adding LST did not substantially improve model fit in the western districts, whereas both LST and moisture variables contributed to model fit in the eastern districts. Overall, the most parsimonious models of late peak season malaria in the western districts were driven by LST alone in the dry and early transition season models and by rainfall alone in the wet season model. In contrast, the best models of late peak season malaria in the eastern districts included a combination of LST and moisture variables in all three seasons (Table 2). In the western districts, the best model based on early transition season climate had a better fit than the best models based on dry and wet season climate as indicated by its lowest AIC value. In the eastern districts, the best models based on dry and early transition season climate both had better fits than the best model based on wet season climate. There was no substantive difference (delta AIC values <2) between the best models based on dry season climate versus early transition season climate in the eastern districts.

Early peak malaria incidence was strongly associated with the subsequent late peak malaria incidence. Spearman’s rank correlation of early and late peak malaria incidence was 0.97 (*p*-value <0.001) for the western districts combined and 0.91 (*p*-value <0.001) for the eastern districts combined. Spearman rank correlations of early and late peak malaria incidence in the individual districts were all positive and were statistically significant (alpha-level of 0.05) in 12 of the 16 districts (Table 3).

**Table 3:** Summary of Spearman rank correlation coefficients between early and late peak malaria incidence for the western and eastern districts

Sub region	District	Correlation
Eastern districts	Artuma Fursi	0.97**
	Bati	0.78**
	Berehet	0.46
	Guba Lafto	0.85**
	Lasta	0.76*
	Merhabete	0.37
	Minjar Shenkora	0.68*
	Sekota	0.87*
	Worebabu	0.62*
Western districts	South Achefer	0.81**
	Ankasha Guangusa	0.63*
	Dembecha	0.86**
	Fagita Lekoma	0.44
	Libokemkem	0.77*
	Mecha	0.93**
	Tach Armacho	0.56

* 0.01 < = *p* < 0.05

** *p* < 0.01

**Fig. 5:**
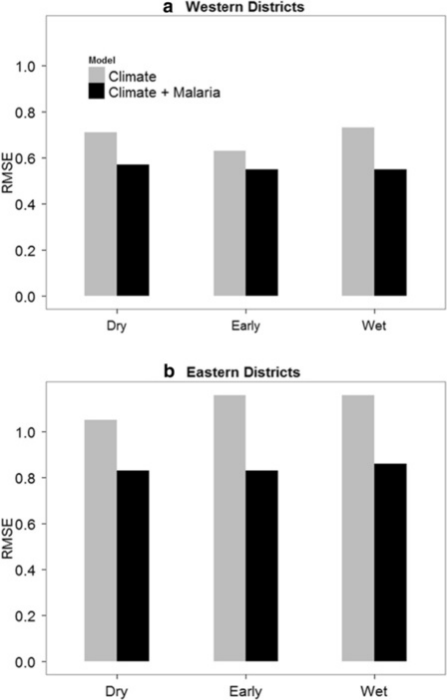
Comparisons of root mean square error (RMSE) for models using climate (*grey*) and climate plus early peak malaria incidence (*black*) to predict late peak malaria incidence in (**a**) Westen districts and (**b**) Eastern districts in the Amhara Region of Ethiopia. RMSE is in units of the natural logarithm of malaria incidence (per 1000)

For late peak season malaria, we compared two GLM models including model 1 (without early peak malaria) and model 2 (with early peak malaria) based on the best model for each season in order to evaluate the influence of early peak malaria incidence on subsequent late peak season epidemics (Table 4). In the western districts, model 2 was the best model as compared to model 1 across all the seasons including the dry (Akaike weight = 1.0), early transition (Akaike weight = 0.99) and wet (Akaike weight = 1.0) seasons. Similarly, in the eastern districts, model 2 was the best model as compared to model 1 across the dry (Akaike weight = 1.0), early transition (Akaike weight = 1.0) and wet (Akaike weight = 1.0) seasons. Additionally, model comparison based on root mean square error (RMSE) showed that model 2 was the best model in all the three seasons across the two regions (Fig. 5). Overall, our results showed that the addition of early peak malaria incidence as an independent variable in model 2 substantially improved model fit in the dry, early transition and wet season models for both regions.

**Table 4:** Best-fitting models predicting late peak season malaria in western and eastern districts

	Season	Model 1 AIC	Model 2 AIC	Delta AIC	Akaike weight model 1	Akaike weight model 2
Western districts	Dry	568.11	549.09	19.02	0.00	1.00
	Early	554.75	543.27	11.48	0.003	0.99
	Wet	572.13	544.18	27.95	0.00	1.00
Eastern districts	Dry	633.98	593.61	40.37	0.00	1.00
	Early	635.95	592.90	43.05	0.00	1.00
	Wet	638.99	600.78	38.21	0.00	1.00

model 1 (without early peak malaria incidence), model 2 (with early peak malaria incidence)

## Discussion

In the Amhara region of Ethiopia, early peak malaria was associated with climate conditions during the preceding dry, and early transition seasons. Moreover, early peak malaria incidence was correlated with malaria incidence during the subsequent late peak season, and malaria incidence during the late peak season had stronger associations with dry, and early transition climate conditions than wet season climate conditions. These results supported a conceptual model of cascading seasonal effects in which the influences of climate on mosquito population dynamics and parasite development are high at the end of the dry season and the beginning of the wet season, and the resulting effects on human disease are then sustained through the wet season and influence malaria incidence during the subsequent late peak season. Further, our results showed that temperature (LST) was the main climatic driver of malaria incidence in the western districts while moisture variables (rainfall, EVI, ET) were important drivers in the eastern districts.

A study in Botswana similarly reported the association of seasonal rainfall (December–February) with malaria incidence during the peak malaria transmission periods (March–April) [37]. The results of our study further suggest that temperature, as well as precipitation, may be an important constraint on malaria at the beginning of the rainy season in highland areas such as the Amhara region. We would expect carryover of malaria transmission from the early to late peak seasons because of long-term asymptomatic carriage of *Plasmodium* parasites in the human population [21–23]. These findings are consistent with earlier work in the western highlands of Kenya that reported strong influences of precipitation on early malaria cases prior to the rainy season, and subsequent association of early peak cases with late peak cases after the rainy season [1]. Our results from the Ethiopian highlands suggest that this type of “ripple effect” may be a more generalized phenomenon of malaria epidemiology in regions characterized by seasonal climates.

Because of these carryover effects, the addition of early peak malaria incidence as an independent variable in models of late peak season malaria improved fit compared with models that used climate variables alone. The association between early and late peak malaria was consistent across models using dry, early transition and wet season climatic factors in both sub-regions. Previous studies also confirmed that the addition of monthly lagged malaria cases to time series models improves prediction of malaria [20, 29]; however, our analysis used malaria data summarized over multiple seasons rather than weekly or monthly time series. This finding emphasizes the importance of accounting for both preceding climate condition and recent trends in malaria indicators in modeling and forecasting efforts. Earlier work has also highlighted the need for integrating early detection (based on disease surveilance) and early warning (based on environmental monitoring) approaches to enhance control and elimination strategies for malaria [20, 30, 38, 39].

In the colder western districts, temperature may limit the growth and biting rates of anopheline mosquitoes and increase the duration of sporogonic cycles of the *Plasmodium* parasites. In contrast, in the drier eastern districts, soil moisture can limit availability of potential breeding sites and thereby limit the development of anopheline larvae and decrease mosquito abundance. These underlying relationships are reflected in the subregional models, in which moisture variables had a stronger relative importance in the eastern districts than in the western districts. These results were also consistent with a previous study in Ethiopia that reported minimum temperature was significantly associated with malaria cases in the cold districts, whereas rainfall was associated with transmission in the hot districts [11]. More generally, multiple studies across Africa and the globe have found that the relative importance of climatic drivers varies geographically depending on the local climate setting, land cover and land use, and hydrology [3, 40, 41]. Thus, our findings support the importance of spatial stratification and the development of forecasting approaches that incorporate spatial heterogeneity in the underlying relationships with climate [40].

Findings from the current study emphasize the importance of understanding both the spatially heterogenous effects of preceding seasonal climatic factors on malaria as well as the potential for carryover of malaria throughout the transmission periods. However, our study has several limitations which may have influenced the results of our analysis. District-level intervention data such as long-lasting insecticide-treated nets, and indoor residual spraying were not available and were not incorporated in our models. As a result of a large scale national control strategy by the government of Ethiopia, approximately 20 million long-lasting insecticide-treated nets (LLINs) have been distributed to all malarious areas all over the country including the Amhara region. Further, the analysis examined associations of climate variables and malaria incidence at the relatively coarse spatial scale of districts ranging from approximately 80 to 2700 km^2^ in size. Yet we expect that there are also more localized ecological and land cover determinants may also influence transmission, and these relationships could be better represented at a finer sub-district scale analysis. We suggest that future studies build on these findings and further assess the associations of seasonal climate variation and malaria by including public health interventions, socioeconomic factors, irrigation and population mobility data at finer spatial scales, including kebele (sub-district administrative unit) and village levels.

## Conclusions

Our study found strong associations of malaria incidence in the early and late peak transmission seasons with preceding climate conditions in the dry, and early transition seasons. Temperature had the strongest influence on malaria incidence in the western districts, whereas moisture variables were more important in the eastern districts. Additionally, our results confirmed strong correlations between malaria incidence in the early and subsequent late peak transmission seasons, and the addition of early peak malaria incidence as an independent variablestrongly improved the fit of the late peak season malaria models. Overall, our findings suggest that associations between the preceding seasonal climate conditions and subsequent malaria transmission during the late peak season may be due to the cumulative effects of climatic factors as well as the potential for parasite carriage across multiple seasons. As a result, forecasting model predictions could be improved by incorporating both preceding climate condition and malaria incidence, which suggests the importance of combining early detection and early warning approaches for malaria epidemic forecasting [42]. Additionally, the finding of different climatic effects on malaria in the different sub-regions emphasizes the the need for spatial stratification and the development of localized models for malaria forecasting.
